# Effect of Spatial and Temporal Prediction on Tactile Sensitivity

**DOI:** 10.3390/brainsci14080749

**Published:** 2024-07-26

**Authors:** Hiroshi Kunimura, Hitoshi Oda, Taku Kawasaki, Han Gao, Shiho Fukuda, Koichi Hiraoka

**Affiliations:** 1 Graduate School of Rehabilitation Science, Osaka Metropolitan University, 3-7-30 Habikino, Habikino City 583-8555, Osaka, Japan; hiroshihayabusa821@yahoo.co.jp (H.K.); t.kawasaki@hakuho.ac.jp (T.K.);; 2 School of Medicine, Osaka Metropolitan University, 3-7-30 Habikino, Habikino City 583-8555, Osaka, Japan

**Keywords:** tactile sensitivity, temporal prediction, spatial prediction

## Abstract

The purpose of the present study was to examine whether spatial or temporal prediction of the tactile stimulus contributes to tactile sensitivity. To investigate the effect of spatial prediction on tactile sensitivity, electrical stimuli were provided for the digit nerve in one of five fingers, and advanced notice of the stimulating finger was provided before the stimulus in some trials but not in others. There was no significant effect of spatial prediction on the intensity at the perceptual threshold of the digit nerve stimulus. This indicates that spatial prediction of the tactile stimulus does not influence tactile sensitivity. To examine the effect of temporal prediction, an auditory warning cue was provided 0, 1, or 10 s before the electrical stimulus to the digit nerve. The stimulus intensity at the perceptual threshold in the trials with the 1 s warning cue was lower than those with the 0 s warning cue. This indicates that temporal prediction enhances tactile sensitivity. The stimulus intensity at the perceptual threshold in the trials with the 1 s warning cue was lower than those with the 10 s warning cue. This means that the contribution of temporal prediction to the tactile sensitivity is greater as the warning cue is closer to the time of the stimulus. This finding may be explained by a defense mechanism activated when humans predict that a tactile stimulus is coming soon.

## 1. Introduction

Humans predict upcoming events to guide perception and action in the process of adaptive behavior. One assumes that any decision, behavior, or perception in humans is based on prediction of a forthcoming event [1]. Especially, there is evidence supporting the view that the prediction of a forthcoming stimulus to the body causes modulation of the perceptual process. For example, aperiodic predictable stimuli increased perceptual sensitivity to a visual cue [2]. Such an effect is also observable for tactile sensitivity. The tactile sensitivity to self-induced tickling was lower than that to the externally induced tickling [3,4]. Those findings have been considered to be due to the prediction of the tactile stimulus mediated by the efference copy of the voluntary movement [5,6,7]. Tactile response accuracy was enhanced by an object coming near the body [8]. Suppression of tactile sensitivity was specifically tuned to the predicted sensory states of a movement [9]. Accordingly, prediction of the tactile stimulus is likely one mental process that tunes the tactile sensitivity.

One aspect of prediction is temporal prediction. Temporal prediction reduced the long-latency motor response to the postural perturbation in stance [10]. Temporal prediction of the choice-reaction time task facilitated the motor processing [11]. Temporal prediction improved the precision of auditory processing [12]. Accordingly, temporal prediction modulates the sensorimotor system. This effect may reflect the influence of the preparatory process of the sensorimotor response to the predicted forthcoming perceptual event. Despite those previous findings, the effect of temporal prediction on tactile sensitivity has not been elucidated.

Another aspect of prediction is spatial prediction. The effect of spatial prediction on tactile sensitivity has not been reported, but that of spatial attention has been reported. Attention to one body part decreased the oxygen blood flow of the body representation in the primary sensory cortex [13]. Tactile selective spatial attention was small or absent; i.e., tactile perception of abrupt texture change was independent of directed attention [14]. Response to the tactile stimulus was facilitated by the prior presentation of the auditory or visual cue given close to the stimulated hand [15,16,17,18], indicating that spatial attention to the body of the stimulated site facilitates the response to the tactile stimulus. Spatial attention must be directed to the forthcoming stimulus when one predicts the site of the stimulus. Thus, spatial attention must be one mental process that occurs with spatial prediction. Accordingly, spatial prediction may influence tactile sensitivity through change in spatial attention. Taken together, we assume that time and/or spatial prediction enhances tactile sensitivity. We examined this hypothesis in the present study.

From a clinical viewpoint, the findings in the present investigation may be beneficial for the treatment of the patients with tactile sensation loss. Patients with diabetes, stroke, or peripheral nerve injury sometimes suffer loss of tactile sensation. It is difficult for the patients to recover from this dysfunction. If the tactile sensitivity is modulated by tactile or spatial prediction, the finding may be a clue for rehabilitation of those patients to recover from this dysfunction.

## 2. Materials and Methods

### 2.1. Participants

Sixteen healthy humans aged 31.3 ± 9.9 years (15 males and 1 female) participated in this study. The sample size was determined by power analysis using G*Power 3.1.9.6. To achieve 80% power with an alpha of 0.05, a total of 12 participants were required to detect a large effect (f = 0.4) for one-way repeated measures analysis of variance (ANOVA) in session 1. To achieve 80% power with an alpha of 0.05, a total of 10 participants were required to detect a large effect (f = 0.4) of interaction between the main effects (prediction and finger) for two-way repeated measures ANOVA in session 2. All the participants were right-handers according to the score by the Edinburgh Handedness Inventory [19]. An experimenter verbally translated English into Japanese for the sentence of each item in this inventory whenever the participants requested. Written informed consent was obtained from the participants. The experiment was approved by the ethics committee of Osaka Metropolitan University (2023-106).

### 2.2. Apparatus

The participants sat on a chair with their arms comfortably beside the trunk. Two ring electrodes providing electrical stimulus were attached to the right thumb (D1), index finger (D2), middle finger (D3), ring finger (D4), and little finger (D5). The distance between the two ring electrodes was 1.5 cm. An anode of the electrodes was placed at the skin over the proximal phalanx and a cathode was placed at the skin over the middle phalanx. The electrical stimuli sent to each finger were produced by an isolator (SS-104J, Nihon Kohden, Tokyo, Japan) connected to a stimulator (SEN-8203, Nihon Kohden, Tokyo, Japan). The duration of the electrical stimulus was 500 μs. Earphones providing a warning cue were placed in the ears, and earmuffs reducing auditory noise were placed over the ears.

### 2.3. Testing Perceptual Threshold

**Figure 1:**
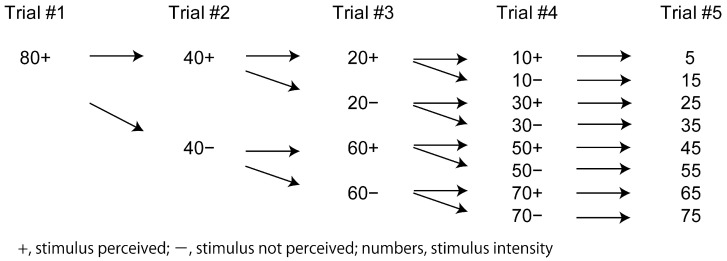
The procedure to determine perceptual threshold. When the participants perceive the stimulus in the first trial, the stimulus intensity in the second trial is half of the intensity (individual approximate threshold intensity). If they perceive the stimulus in the second trial, the intensity in the third trial is half of the stimulus intensity of the second trial. If they do not perceive the stimulus in the second trial, the stimulus intensity in the third trial is the mid-value of the intensity between the first and second trials. This procedure continues until the minimum intensity at which the participants perceive the stimulus (individual precise threshold) is determined.

Several trials were conducted in a trial set to determine the perceptual threshold to the digit nerve stimulus in each participant. The stimulus intensity at the perceptual threshold was determined via a “method of adjustment” in which the minimum intensity at the perceptual threshold was searched by increasing and decreasing the stimulus intensity [20]. An example of the procedure in a trial set is shown in Figure 1. The individual approximate stimulus intensity at which each participant perceived the stimulus (individual approximate perceptual threshold) was estimated via a rapid increase in the stimulus intensity from trial to trial before beginning each session (a session investigating the effect of time prediction and another session investigating the effect of spatial prediction). The stimulus intensity in the first trial of each trial set was at the intensity of the individual approximate perceptual threshold. They perceived the stimulus in the first trial because the individual approximate threshold determined via the fast increase in the stimulus intensity from trial to trial was always above the individual precise perceptual threshold. Once they perceived the stimulus in the first trial, the stimulus intensity in the second trial was half of the intensity in the first trial. If they perceived the stimulus in the second trial, the stimulus intensity in the third trial was half of the intensity in the second trial. If they did not perceive the stimulus in the second trial, the stimulus intensity in the third trial was the mid-value between the intensity of the first and second trials. This procedure continued until the minimum intensity at which the participants perceived the stimulus (individual precise perceptual threshold) was determined.

### 2.4. Temporal Prediction Session

**Figure 2:**
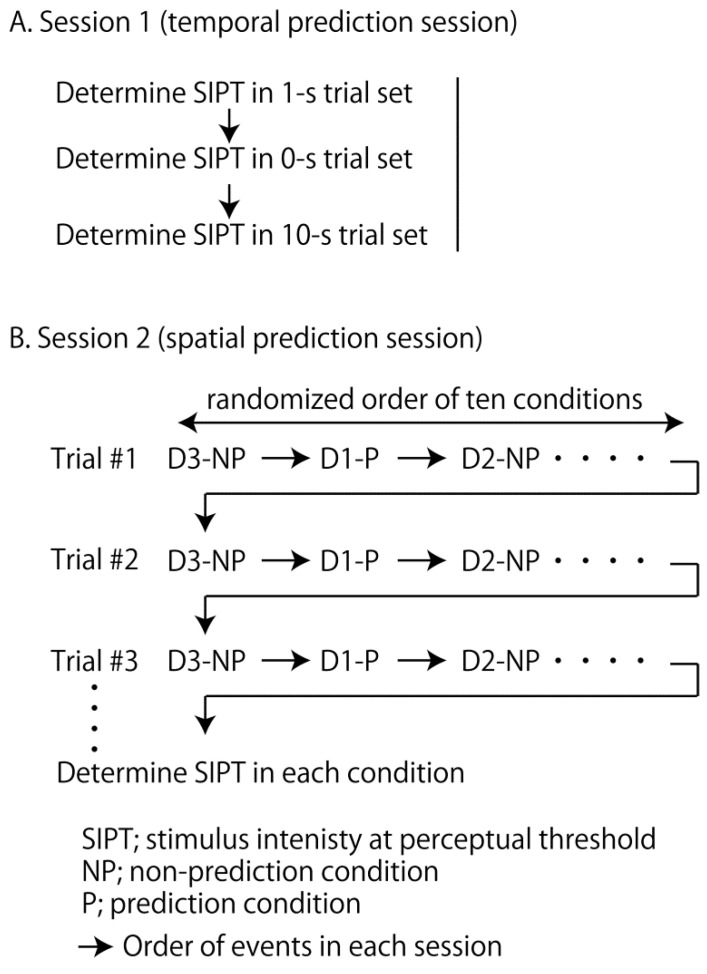
The procedure in session 1 (**A**) and 2 (**B**). Each trial set is conducted independently in session 1 (**A**). All of ten conditions are conducted in each trial number until the stimulus intensity at the perceptual threshold (SIPT) in each condition is determined in session 2 (**B**).

In session 1, the effect of temporal prediction on the tactile sensitivity to the digit nerve stimulus was examined (temporal prediction session). The procedure of this session is shown in Figure 2A. Electrical stimuli were provided to D2. D2 was tested because many previous studies found a significant effect of the conditioning stimulus on the tactile sensitivity of D2 [21,22]. The participants answered “yes” when they perceived the stimulus. In the 1 s warning trial set, an auditory warning cue was provided 1 s before each stimulus. In the 10 s warning trial set, the cue was provided 10 s before the stimulus in each trial. In the 0 s warning trial set, the cue was provided simultaneously with the stimulus. Different intervals between the warning cue and stimulus were assigned in each trial set to examine the effect of temporal prediction on the perceptual threshold to the digit nerve stimulus. The intervals of 0, 1 and 10 s were used because the warning cue given 5 s before the perturbation was effective on the motor response to the postural perturbation but that given 1 or 3 s before the perturbation was not effective [10], indicating that temporal prediction changes in the time between 1 and 5 s after the onset of this process. Each of three trial sets was conducted independently (Figure 2A). To test the perceptual threshold without the influence of the drift of the perceptual threshold over time, the order of these three trial sets was assigned pseudo-randomly to each participant.

### 2.5. Spatial Prediction Session

In session 2, the effect of spatial prediction on the tactile sensitivity to the digit nerve stimulus was examined (spatial prediction session). The procedure of this session is shown in Figure 2B. Electrical stimuli were provided to D1, D2, D3, D4, or D5 in each trial in a pseudo-random manner. The participants answered the finger that they perceived. If they did not answer the perceived finger or indicated the incorrect finger, the stimulus intensity in this trial was considered to be below the threshold. The notice of the finger to be stimulated was given to the participants before beginning the predictable trial. The experimenter did not inform the finger to be stimulated in the non-predictable trial. The predictable and non-predictable trials were randomly assigned in each trial. Taken together, one of ten conditions (i.e., five digits for two spatial predictability conditions) was assigned to each trial, and the trials were repeated until the precise stimulus intensity at the perceptual threshold was determined in all the ten conditions. The order of the stimulated fingers and prediction conditions was randomized to test the perceptual threshold without the influence of the drift of the perceptual threshold over time.

### 2.6. Statistical Analysis

One-way repeated measures analysis of variance (ANOVA) was conducted to test the effect of the warning cue (0 s, 10 s, and 1 s warning cues; 3 levels) on the perceptual threshold for the digit nerve stimulus in session 1. Two-way repeated measures ANOVA was conducted to test the main effect of the stimulated finger (D1, D2, D3, D4, and D5; 5 levels) and that of the spatial prediction (predictable and non-predictable; 2 levels) on the perceptual threshold for the digit nerve stimulus in the session 2. The result of Greenhouse–Geisser’s correction was reported whenever Mauchly’s test of sphericity was significant. A multiple comparison test (Bonferroni test) was conducted when the main effect or simple main effect was significant. The alpha level was 0.05. Statistical analyses were carried out using Excel Tokei ver. 3.20 (Social Survey Research Information, Tokyo, Japan). We used means and standard errors of means across participants to describe the results.

## 3. Results

**Figure 3:**
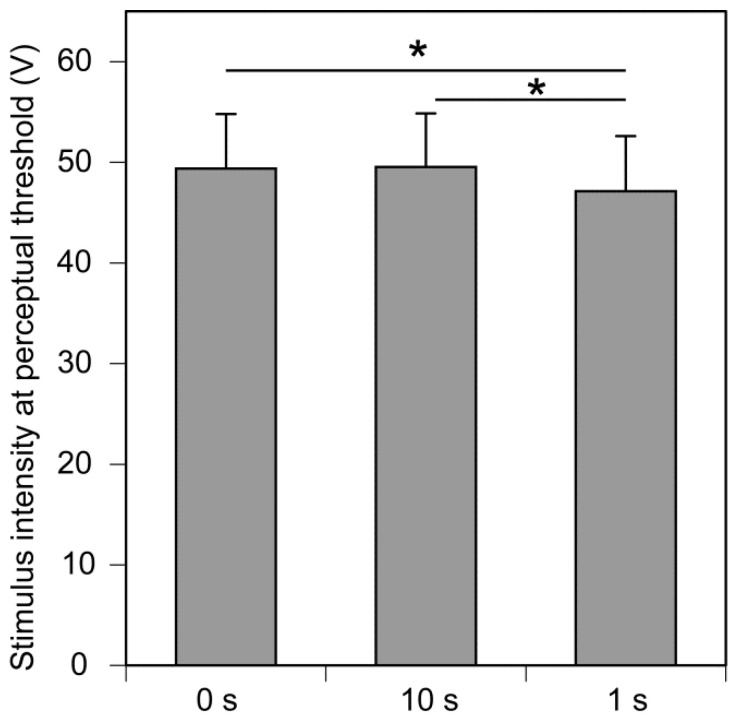
The effect of time prediction on the stimulus intensity at the perceptual threshold. Bars indicate the mean and error bars indicate the standard error of the mean. Asterisks indicate a significant difference (*p* < 0.05).

The results of session 1 investigating temporal prediction are shown in Figure 3. The ANOVA revealed that the main effect of temporal prediction on the stimulus intensity at the perceptual threshold was significant [F(2, 30) = 4.687, *p* = 0.017, η^2^p = 0.238]. Multiple comparison tests revealed that the stimulus intensity at the perceptual threshold in the 1 s warning trial set was significantly lower than that in the 10 s (*p* = 0.032) and 0 s warning trial sets (*p* = 0.047). There was no significant difference in the stimulus intensity at the perceptual threshold between the 0 s and 10 s warning trial sets (*p* = 1.000).

**Figure 4:**
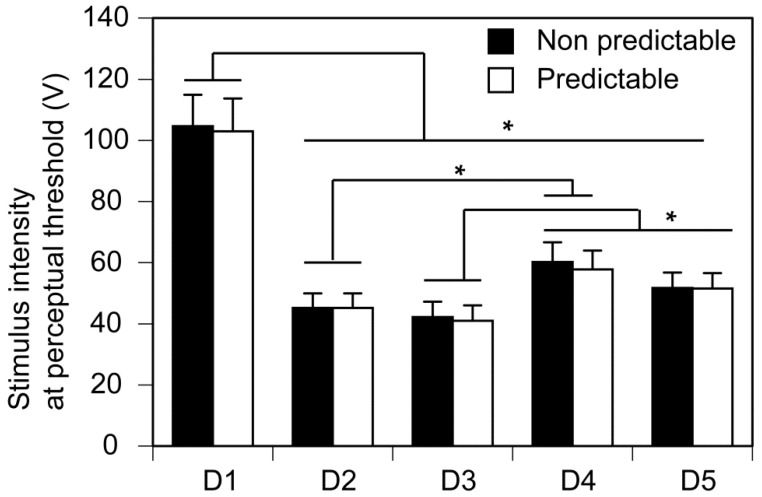
The effect of spatial prediction on the stimulus intensity at the perceptual threshold. Bars indicate the mean, and error bars indicate the standard error of the mean. Asterisks indicate a significant difference between the fingers (*p* < 0.05).

The results of session 2 investigating the effect of spatial prediction are shown in Figure 4. One participant dropped out of the experiment after the completion of session 1 due to fatigue. Thus, the number of the participants in session 2 was 15. There was no significant interaction between the two main effects [F(4, 56) = 0.428, *p* = 0.788, η^2^p = 0.030]. There was a significant main effect of the stimulated finger on the stimulus intensity at the perceptual threshold [F(4, 56) = 61.588, *p* < 0.001, η^2^p = 0.815]. The stimulus intensity at the perceptual threshold of the stimulus in D1 was significantly greater than the other fingers (*p* < 0.05). The stimulus intensity at the perceptual threshold of the stimulus in D2 was significantly lower than that in D4 (*p* < 0.05). The stimulus intensity at the perceptual threshold of the stimulus in D3 was significantly lower than that in D4 or D5 (*p* < 0.05). There was no significant main effect of spatial prediction on the stimulus intensity at the perceptual threshold [F(1, 14) = 3.312, *p* = 0.090, η^2^p = 0.191].

## 4. Discussion

In the present study, an investigation was made to determine whether spatial or temporal prediction of the tactile stimulus contributes to tactile sensitivity. There was a significant effect of temporal prediction on the perceptual threshold of the digit nerve stimulus. In contrast, there was no significant effect of spatial prediction on that.

### 4.1. Temporal Prediction

The perceptual threshold in the 1 s warning trial set was lower than that in the 0 s trial set. The participants could not predict the time of the stimulus because the stimulus and warning cue were provided simultaneously in the 0 s trial set. In contrast, in the 1 s trial set, they could predict the time of the stimulus because the warning cue was provided immediately before the stimulus. The digit nerve purely mediates the cutaneous sensation. Thus, the finding indicates that temporal prediction of the tactile stimulus enhances tactile sensitivity.

Regarding the significant difference in the stimulus intensity at the perceptual threshold between the 1 s and 0 s trial sets in session 1, one possible view must be noted. A warning sound cue was provided simultaneously with the tactile stimulus to the D2 in the 0 s trial set. One possible interpretation of this finding is that the warning sound cue interfered with the perception of the tactile stimulus when the cue was delivered simultaneous with the tactile stimulus. That is, this sound cue may have masked the tactile perception. This explanation may be possible for the greater tactile sensitivity in the 1 s trial set compared with that in the 0 s trial set. However, there is a contradictory view of this interpretation. When two different modalities of the stimuli are provided simultaneously, the motor execution process is facilitated (i.e., reaction time is shortened), called intersensory facilitation [23]. Perception of the imperative cue (stimulus) and motor execution are involved in the reaction time. Thus, intersensory facilitation may be explained by the facilitation of the perceptual process of the stimulus. Accordingly, further studies are needed to rule out the possible view that the warning sound cue provided simultaneously with the tactile stimulus masks the tactile perception.

Time prediction influences the motor response to postural perturbation. A warning cue indicating the onset of the postural perturbation in stance provided 2 s before the perturbation induced the cortical preparatory activity and decreased the displacement of the center of pressure during the perturbation [24,25]. Thus, the effect of temporal prediction on the motor response to the perturbation is due to the preparatory activity of the motor response to the postural perturbation at the cortical level.

A warning cue provided 5 s before the perturbation attenuated the long-latency motor response of the ankle extensor to the postural perturbation in stance, but it did not when the cue was provided 1 or 3 s before the perturbation [10]. This finding was interpreted by the view that temporal prediction takes 5 s or more to be effective for the motor response process to the postural perturbation. In contrast, the perceptual threshold in the 1 s warning trial set was lower than that in the 10 s warning trial set in the present study. This means that the tactile sensitivity was greater as the time between the warning cue and stimulus decreased. Those conflicting findings between the previous study of the motor response to the postural perturbation and the present study of the tactile sensitivity are explained by the view that significant time is needed to be effective for the preparation of the response to perturbation by the prediction of the perturbation time, but the tactile sensitivity is enhanced as humans predict that a tactile stimulus is coming soon.

Acuity of the response to the tactile stimulus was enhanced when one perceived an object coming near the body [8]. The ankle muscle activity tended to be greater as the time to stimulate the head got closer [26]. Those previous findings are explained by defense mechanisms to block the stimulus or to keep the body away from the stimulus [27]. The withdrawal reflex elicited by a noxious tactile stimulus and the startle response induced by the loud auditory stimulus are typical defense responses in humans [28,29,30,31]. Such a defense mechanism was activated even when the tactile stimulus was provided to the head; when a tactile stimulus (air puff) was given to the body, monkeys moved their heads away from the stimulus [32].

In the present study, the tactile sensitivity increased as the time of the warning cue got close to the time of the tactile stimulus. This is interpreted by the view that humans increase tactile sensitivity when they predict that the tactile stimulus is coming soon. This may represent a defense mechanism activated when they predict that the tactile stimulus is coming soon.

An alternative explanation may be possible for the present finding. The difference between the tactile perceptual sensitivity when the warning cue was provided 1 s before the stimulus and the sensitivity when the cue was provided 10 s before that may have been related to the decay of short-term memory over time. Short-term memory decays over time [33,34]. Thus, memory of the warning cue at the moment at which the participants answer the perception of the tactile stimulus is more greatly decayed when the warning cue was provided 10 s before the stimulus, causing the smaller effect of the warning cue on tactile sensitivity. Further studies are needed to rule out this alternative view.

### 4.2. Spatial Prediction

There was no significant effect of spatial prediction on the stimulus intensity at the perceptual threshold. This indicates that spatial prediction does not influence the perceptual threshold of the digit nerve stimulus. In the present study, spatial prediction did not indicate that the stimulus is close to the body. Rather, the warning cue just indicated where the stimulus was going to be provided. Thus, the negative finding may be explained by the view that spatial prediction induced by the warning cue did not indicate whether the stimulus was close to the body influencing the defense mechanism.

### 4.3. Limitations

In the present study, the number of female participants was only one, although about half of humans are female. There is a gender difference in tactile perceptual threshold [35,36,37,38]. Accordingly, the present finding may be influenced by the deviation of the sample size between the males and females. In future studies, the effect of the prediction on tactile sensitivity must be investigated in the female group.

The number of participants in the present study was 16. Twelve was the typical number of participants in previous studies investigating the tactile sensitivity [39,40]. According to power analysis, the number of participants required to detect a large effect of interaction between the two main effects was 12. In session 2 (spatial prediction session), a significant interaction between the main effects was not revealed. The sample size for detecting a medium effect is 22 according to power analysis. Thus, in session 2, there is a possible view that the insignificant interaction between the main effects is caused by too small sample size to detect a medium effect. 

## 5. Conclusions

The stimulus intensity at the perceptual threshold in the trials with a warning cue 1 s before the stimulus was lower than that with the cue provided simultaneously with the stimulus. More importantly, the stimulus intensity at the perceptual threshold in the trials with a warning cue 1 s before the stimulus was lower than that with a warning cue 10 s before the stimulus. This means that the contribution of temporal prediction to tactile sensitivity is greater as the warning is closer to the time of the stimulus. The explanation for this finding is that the defense mechanism to the tactile stimulus is activated when humans predict that it is coming soon. This is a pilot study, and thus, further investigations are needed for further insight into the effect of temporal and spatial prediction on tactile sensitivity.

## Data Availability

The datasets used or analyzed in this study can be obtained from the corresponding author upon reasonable request. The data are not publicly available due to privacy issues.
